# Proteomic analysis of saliva: a unique tool to distinguish primary Sjögren's syndrome from secondary Sjögren's syndrome and other sicca syndromes

**DOI:** 10.1186/ar3523

**Published:** 2011-11-25

**Authors:** Chiara Baldini, Laura Giusti, Federica Ciregia, Ylenia Da Valle, Camillo Giacomelli, Elena Donadio, Francesca Sernissi, Laura Bazzichi, Gino Giannaccini, Stefano Bombardieri, Antonio Lucacchini

**Affiliations:** 1 Department of Internal Medicine, Rheumatology Unit, University of Pisa, Via Roma 67, 56126 Pisa, Italy; 2 Department of Psychiatry, Neurobiology, Pharmacology and Biotechnology, University of Pisa, Via Bonanno 6, 56126, Pisa, Italy

**Keywords:** proteomics, whole saliva, primary Sjögren's syndrome, secondary Sjögren's syndrome

## Abstract

**Introduction:**

A growing interest has arisen in salivary proteomics as a tool for the identification of biomarkers for primary Sjögren's syndrome (pSS). Nonetheless, only a limited number of preclinical validation studies have been performed, limiting the possibility of translating proteomic results into clinical practice. The primary aim of this study was to refine the diagnostic power of a panel of candidate salivary biomarkers described in pSS with respect to both healthy volunteers and pathological controls. We also explored the pathogenetic function of the detected putative biomarkers both in the local exocrinopathy and in the systemic inflammatory processes of SS.

**Methods:**

One hundred and eighty patients were included in the study overall. In the first "exploratory phase", we enrolled 40 females with pSS, 40 sex- and age-matched healthy volunteers, 10 patients with sicca non-SS and 15 secondary SS (sSS) patients. The testing cohort of the second "challenge phase" of the study was represented by 75 unselected, consecutive subjects: 19 pSS, 21 healthy volunteers, 10 sicca non-SS and 25 sSS patients. Salivary proteomic analysis was performed combining two-dimensional electrophoresis (2DE) and matrix-assisted laser desorption/ionisation time-of-flight mass spectrometry (MALDI-TOF-MS). Western blot (WB) analysis and enzyme-linked immunosorbent assay (ELISA) were employed to validate 2DE results. Ingenuity Pathway Analysis (IPA) Knowledge base was adopted to associate candidate biomarkers in a signalling pathogenetic network.

**Results:**

A total of 28, 6, 7 and 12 protein spots were found to be significantly different in pSS samples with respect to healthy volunteers, non-SS sicca syndrome, SSc-sSS and rheumatoid arthritis-sSS, leading to the identification of 15 differently expressed proteins. Among them, α-amylases precursor, carbonic anhydrase VI, β-2 microglobulin, glyceraldehydes-3-phosphate dehydrogenase (G3PDH), epidermal fatty acid binding protein (E-FABP) and immunoglobulin k light chain (IGK-light chain) apparently showed the most significant differences in pSS when compared to healthy volunteers and non-SS pathological controls. On the other hand, as expected, pSS and sSS salivary profiles shared a great number of similarities.

**Conclusions:**

This study demonstrated that salivary fluid might represent a novel ideal milieu for the detection of a diagnostic panel of candidate biomarkers for pSS, and to gain an insight into the pathogenetic processes underlying glandular and systemic autoimmune disorders.

## Introduction

Sjögren's syndrome (SS) is an autoimmune exocrinopathy characterised by the infiltration of salivary and lacrimal glands by mononuclear cells with secondary destruction of the parenchymal tissue, resulting in oral and ocular dryness [1,2]. Several glandular and extraglandular manifestations may be part of the full spectrum of the disease, which might severely affect the patients' prognosis and quality of life [3-7]. The complexity of SS clinical presentation is increased by the fact that SS may occur alone, as a primary condition (primary Sjögren's syndrome-pSS), or in association with other connective tissue diseases, including rheumatoid arthritis (RA) and systemic sclerosis (SSc) as secondary SS (sSS) [8-10].

In order to improve the diagnostic algorithm for pSS, during the last few years, growing interest has been raised for salivary proteomics as a promising tool for the discovery of disease biomarkers both for pSS and for other autoimmune and non-autoimmune disorders [11-17]. In particular, saliva, which may closely reflect the underlying pathogenetic process in salivary glands, has been viewed as an attractive biofluid for proteomic research in pSS and a number of studies have so far outlined the potential differences between pSS and healthy controls [11-17]. In 2007, we also carried out a pilot study aimed at characterising the salivary proteomic profile of 12 pSS patients in comparison to 12 sex- and age-matched healthy individuals [12]. By using quantitative two-dimensional electrophoresis (2DE) and matrix-assisted laser desorption/ionisation time-of-flight mass spectrometry (MALDI-TOF-MS), we identified 15 differently expressed proteins which apparently reflected the various histopathological aspects of pSS, from acinar loss, to lymphocytes infiltration, to local and systemic flogosis. At the time, the pattern of identified proteins was not preclinically validated.

The aim of the current study was, therefore, to analyse by mass spectrometry techniques, coupled with Western blot (WB) and enzyme-linked immunosorbent assay (ELISA), the proteomic profile of pSS in an independent larger cohort of patients not only in comparison to healthy volunteers but also in comparison to pathological controls. To this purpose, we included subjects with non-SS sicca syndrome which may provide a natural model of chronic dryness of the oral cavity not sustained by an autoimmune response. Moreover, in order to verify whether salivary proteomics might be utilised to distinguish pSS from sSS the study was also extended to patients affected by SS and concomitant RA (RA-sSS) and SSc (SSc-sSS). The ultimate goal of this part of the study was, in other words, to support the work hypothesis that proteomic analysis of whole saliva could represent a novel technique not only for the diagnosis of disorders involving salivary glands but also systemic autoimmune disorders even in the absence of any salivary exocrinopathy.

Finally, we also explored the biological and pathogenetic function of the detected putative discriminatory salivary proteomic biomarkers both in the local exocrinopathy and in the systemic inflammatory autoimmune systemic processes of pSS by employing the Ingenuity Pathway Analysis (IPA) Knowledge base.

## Materials and methods

### Study design

This diagnostic case-control study was subdivided into three different steps. The first "exploratory" phase was aimed at characterising the salivary proteomic profile of a large group of pSS subjects in comparison to healthy controls and pathological controls. The second "challenge" phase was aimed at preclinically validating by WB and ELISA the ability of these candidate biomarkers to differentiate pSS from healthy volunteers, subjects with non-pSS sicca syndrome and patients with sSS. ELISA results were also correlated to the minor salivary gland focus score and to rheumatoid factor, anti-Ro/SSA and anti-La/SSB. Finally, to investigate the biological function of the significantly changing proteins, we applied the Ingenuity Pathway Analysis (IPA) Knowledge base (Ingenuity^® ^System Inc., Redwood City, CA, USA).This platform enabled us to visualize the potential interactions between the identified biomarker signatures in saliva and other molecules of interest, which may not have been detected in this particular study, and to identify biological pathways underlying pSS disease process. The study was approved by the local Ethics Committee (Comitato per la sperimentazione clinica dei farmaci, Azienda Ospedaliera Universitaria Pisana; reference number 3062).

### Patients' inclusion and exclusion criteria

Patients with a diagnosis of pSS made according to the International Classification Criteria for the disease (AECG) [9], and healthy subjects with similar mean age, and demographic characteristics were enrolled. The study was also extended to pathological controls including patients with non-SS sicca syndrome and patients with RA-sSS and SSc-sSS. Similar to pSS, the diagnosis of sSS was made according to the AECG classification criteria [9] while the condition of non-SS sicca syndrome was defined as the presence of xerostomia and xerophtalmia in patients with negative non-organ specific autoantibodies and negative minor salivary gland biopsy (according to the Chisolm and Mason scoring system).

Detailed medical charts were available for all patients. Variables analysed in the study included: sex, date of diagnosis, date of salivary sample collection, disease duration, presence of dry eyes and dry mouth, past or present parotid enlargement and signs or symptoms suggestive for any current or past extra-glandular involvement (arthritis, lung involvement, skin vasculitis, peripheral nervous involvement, kidney involvement). Patients' treatments were also recorded. Moreover, at the time of the study entry, every patient had a complete ophthalmological examination (including Schirmer's test and Green Lissamine test), complete laboratory tests, and an evaluation of the autoantibodies profile (that is, antinuclear antibodies, extractable nuclear antigen (ENA) antibodies, rheumatoid factor, anticentromere auto-antibodies (ACA), Scl-70, anti-cyclic citrullinated peptide (anti-CCP)). As far as laboratory tests were related, a white blood cell count <4,000/mm3, C3 <80 mg/dl, C4 <10 mg/dl, gamma-globulin >1.7 g/dl, creatinine clearence <60 ml/minute, proteinuria >300 mg/day and urinary pH >6 were considered abnormal. Antinuclear antibodies, ACA and Scl-70 antibodies were assessed by indirect immunofluorescence, anti Ro/SSA, La/SSB antibodies by controimmunoelectrophoresis, rheumatoid factor by nephelometry and anti-CCP by ELISA. A minor salivary gland biopsy was performed in all cases. HIV-1, hepatitis B and hepatitis C infections were also excluded in all of the participants. Written informed consent was obtained from all patients and healthy volunteers for their inclusion in the study.

### Saliva collection and processing

Unstimulated whole saliva (WS) samples were collected early in the morning in standard conditions, and the preparation of the samples was performed as previously described [12]. In order to minimize the degradation of the proteins, the samples were processed immediately and kept on ice during the process. Samples were immediately centrifuged at 14,000 g for 30 minutes at 4°C to remove debris and cells, and protein amounts of resulting supernatants were determined using the Bio-Rad protein assay. Aliquots of the samples were stored at -80°C until analysis.

### 2DE analysis

The saliva specimens for each class were pooled and each sample contributed to make the pool with an equal amount of protein. We decided to carry out our study on pooled samples due to the fact that the pool allowed us to eliminate the intra-class variation even if the response of single patients was lost. Salivary proteins (150 µg) were solubilised in rehydration solution (7 M urea, 2 M thiourea, 4% 3-((3-cholamidopropyl) dimethylammonio)-1-propanesulfonate (CHAPS), 60 mM dithiothreitol (DTT), 0.5% 3 to 10 ampholytes and 0.002% of bromophenol blue). 2DE was performed using the Immobiline-polyacrylamide system as previously described [12]. Analytical gels were silver stained [18], and preparative gels were stained using the Blue silver protocol [19]. The 2-DE experiments were performed in triplicate for each pool. No significant quantitative differences among the replicates for each pool were observed. The stained gels were scanned using an Epson Expression 1680 Pro scanner (Seiko Epson Corporation, Nagano, Japan ] and the images were analysed using Image-Master 2D Platinum 6.01 (GE Health Care Europe, Uppsala, Sweden). After comparison of pSS with respect to sSS and other sicca syndromes, proteins whose expression showed over 1.5-fold statistical significant spot quantity change were selected and identified.

### In-gel digestion and mass spectrometry analysis

Spots of interest were cut out from the master gel and de-stained by washing them with 50% acetonitrile in 50 mM ammonium bicarbonate for 30 minutes. Gel pieces were then dried for 30 minutes in a Hetovac vacuum centrifuge (HETO, Allerod, Germany). Dried pieces of gel were subjected to protein digestion by trypsin and peptide extraction. MS and MS/MS analysis of peptides from 2-DE gel spots were performed with a 4800 Proteomics Analyzer MALDI-TOF/TOF mass spectrometer (Applied Biosystems, Framingham, MA, USA) according to the tuning procedures suggested by the manufacturer. Peak lists were generated with the Launch peak to MASCOT tools. Such acquired MS and MS/MS data were compared to the database using the MASCOT search engine [20]. In MASCOT, the combined peptide mass fingerprint and MS/MS search was performed on human entries present in Uni- ProtSPTR database. Search settings allowed one missed cleavage with the trypsin enzyme selected, one fixed modification (carboxymethylated cysteine) and one variable modification (oxidation of methionine).

Scaffold (version Scaffold_2_03_01, Proteome Software Inc., Portland, OR, USA) was used to validate MS/MS based on peptide and protein identifications. Peptide identifications were accepted if they could be established at greater than 95.0% probability as specified by the Peptide Prophet algorithm [21]. Protein identifications were accepted if they could be established at greater than 95.0% probability and contained at least two identified peptides. Protein probabilities were assigned by the Protein Prophet algorithm [22]. Proteins that contained similar peptides and could not be differentiated based on MS/MS analysis alone were grouped to satisfy the principles of parsimony.

### Principal component analysis of match set data

In order to identify patterns in the salivary protein profiles of the different patients' groups, and to express the data in such a way as to highlight their similarities and differences, a mathematical procedure, the principal component analysis (PCA), was applied to the entire data of the match sets, including healthy, pSS, non-SS sicca syndrome and RA-sSS and SSc-sSS subjects. Normalised spot data were imported from an Excel datasheet into the SIMCA-P+12 (Umetrics, Umeå, Sweden) including the density data (% of volume) of about 700 spots/gel to observe the similarity among different classes. To illustrate the arrangement of the clusters produced by hierarchical clustering a tree diagram (dendogram) was produced displaying the results of the cluster analysis.

### Western blot (WB) analysis of the expression of β-2 microglobulin, α-enolase, immunoglobulin k light chain (IGKC) and α-amylase fragmentation pattern

The WB was used to ensure the reliability of the 2DE results. In particular, we investigated the expression of β-2 microgloblulin, α-enolase, immunoglobulin k light chain (IGKC) and the α-amylases. In order to characterise the fragmentation pattern of α-amylases, 2D blots were performed using specific antibody direct *versus *the full length of human recombinant protein in all the groups. In the process of 1D WB, aliquots of samples (pSS, RA-sSS, SSc-sSS, sicca) were mixed with a SDS sample buffer (Laemmly solution) and heated at 100°C for five minutes. The WB was carried out as exactly previously described [17]. Briefly, aliquots of proteins (25 μg), extracts from mix pooled WS samples of each class, were loaded on 12% acrylamide gels and processed [23]. For the protein detection the following antibodies were used: mouse monoclonal anti full length β-2 microgloblulin (sc-58903, Santa Cruz Biotechnology, Santa Cruz, CA, USA), mouse monoclonal anti-α-enolase (sc-100812, Santa Cruz Biotechnology), duck polyclonal anti full length α-amylases (ab31147, Abcam, Cambridge, UK) and rabbit polyclonal anti-IGKC (ab103827, Abcam, UK) All the experiments were carried out in triplicate. For each tested protein, the optical density of specific immunoreactive bands was determined (Image J software RSB, NIH, Betesda, Maryland, USA), and the resulting mean values ± SD were compared.

For the detection of α-amylases, we chose a specific antibody direct versus a full length of human recombinant albumin, to detect the potential protein fragmentation. Aliquots of 100 µg of proteins were separated by 2DE using 3 to 10 linear strips 13 cm before Western blot analysis. The dilution was 1:500 and 1:10,000 for anti-α-amylases primary antibody and anti-duck, respectively.

To assure a correct control of development conditions a solution of standard proteins was loaded in each experiment and the time and temperature of the process were controlled.

### ELISA

ELISA (E90260Hu, E961449Hu, Uscnk, Life Science Inc. ,Wuhan, P.R.China ) were used to determine the level both of β-2 microglobulin and α-enolase in saliva samples from healthy (n = 21), pSS (n = 19), non-SS sicca syndrome (n = 10) and sSS (n = 25) subjects. Whole saliva samples were thawed, vortexed and centrifuged to eventually remove mucins precipitation prior to assaying. Saliva samples were diluted with 20 mM PBS buffer, pH 7.2 at a final dilution of 1:100 and 1:5 for β-2 microglobulin and α-enolase, respectively. The assay was performed according to the manufacturer's instruction manual. Samples were analysed in duplicate, and the protein levels were determined according to the calibration curves established from standards (detection range 0.3 to 25 ng/ml).

### Salivary α-amylase assay

For α-amylase a salivary α-amylase assay kit specifically designed and validated for the kinetic measurement of salivary α-amylase activity was used (catalog No 1-1902, Salimetrics, LLC, State College, PA, USA). Saliva samples were diluted with α-amylase diluent provided at a final dilution of 1:200. Aliquots of 8 μl of diluted samples were added to individual wells and the reaction was started by the simultaneous addition of preheated (37°C) α-amylase substrate solution. The enzymatic activity of α-amylase on chromagenic substrate was determined according to the manufacturer's instruction manual at 405 nm. Results are expressed in U/mg of proteins. The Salimetrics' high and low salivary α-amylase controls were run with each assay.

### Statistical analysis

The optical density of the proteins was expressed as a percentage of the volume (mean ± SD). The significance of the differences (*P*-value <0.05) was calculated using the ANOVA t-test and the Mann-Whitney test according to data distribution. A multivariate analysis of expression data of matched spots was conducted for all classes and evaluated by principal component analysis (PCA). The PC software SIMCA-P+12 (Umetrics, Umeå, Sweden) was used in the PCA analysis.

### Signalling pathways analysis

Functional pathway and network analyses were generated using the IPA software v7.1 (Ingenuity^® ^System). IPA identified the pathways from the IPA library of canonical pathways that were most significant to the data set. Proteins that met the expression ratio with a cut-off ≥2.0, a *P-*value cut-off <0.05 for differential expression and were associated with a canonical pathway in the IPA Knowledge Base, were considered for the analysis. Since one protein may have multiple functions, we selected the functions with *P-*value <0.015. The network proteins associated with biological functions and/or diseases in the IPA Knowledge Base were considered for the analysis. These networks are scored for degree of relevance with values >3 having a 99.9% confidence level of not being generated by random chance alone. The genetic networks that were created describe functional relationships between gene products based on known associations in the literature.

## Results

### Patients

One hundred and eighty subjects were included in the study: 105 subjects were enrolled in the first "exploratory" phase and 75 in the second "challenge" phase. That is, in the first part of the study, we included 40 women with a diagnosis of pSS made according to the International Classification Criteria for the disease [9], 40 healthy age- and sex-matched women, 10 patients with non-SS sicca syndrome, and in 15 patients with sSS: 8 with RA-sSS, and 7 with SSc-sSS. Table 1 summarises the demographic and clinico-serological features of this training set of patients.

**Table 1:** Primary, secondary SS and non-SS sicca patients' demographic and clinical features

Data	pSS (40)	Sicca non-SS (10)	RA-sSS (8)	SSc-sSS (7)
Sex	40 F:0M	10 F:0M	8 F:0 M	7F:0M
Age (yrs)	52.4 ± 10.5	56.0 ± 10.7	60.8 ± 11.4	61.7 ± 8.1
Disease duration (yrs)	7 ± 6	3 ± 2	9 ± 7	7 ± 4
Oral symptoms	98%	80%	100%	86%
Ocular symptoms	97%	80%	100%	100%
Ocular tests (1)	100%	70%	100%	100%
Difficult in swallowing	19%	2%	12%	71%
Salivary gland enlargement	32%	none	12%	none
Minor salivary gland biopsy grading:				
Chisolm and Mason grade 0, 1, 2	None	10/10	5/8	1/7
Chisolm and Mason grade 3	20/40	none	3/8	5/7
Chisolm and Mason grade 4	20/40	none	none	1/7
Arthralgia	76%	1%	100%	57%
Arthritis	6%	none	100%	none
Fatigue	73%	30%	25%	29%
Raynaud's phenomenon	43%	2%	12%	100%
Skin involvement	16%	none	37%	14%
Dyspareunia	32%	2%	none	14%
Interstitial lung disease	None	none	12%	14%
Peripheral nervous involvement	3%	none	12%	none
Kidney involvement	3%	none	none	none
Leucopenia	30%	none	12%	14%
Low C3 and/or C4 levels	33%	1%	12%	14%
Hypergammaglobulinemia	62%	none	37%	none
Antinuclear antibodies	94%	90%	62%	100%
Anti-Ro/SSA	70%/	none	25%	14%
Anti-La/SSB	36%	none	12%	none
Rheumatoid Factor	80%	2%	100%	29%

(1), Schirmer's test and/or Lissamine Green; pSS, primary Sjögren's syndrome; RA-sSS secondary Sjögren's syndrome concomitant to rheumatoid arthritis; SS: Sjögren's syndrome; SSc: systemic sclerosis; SSc-sSS secondary Sjögren's syndrome concomitant to systemic sclerosis; sSS: secondary Sjögren's syndrome

Patients affected by RA-sSS presented a median Disease Activity Score 28 (DAS28) of 3.8 ± 1.2. Seven of them were anti-cyclic citrullinated peptide (CCP) antibodies positive and six out of eight showed the presence of bone erosions on X-ray. At the time of the enrolment in the study, all the RA-sSS were on stable low-dose prednisolone associated to biological or non biological disease-modifying antirheumatic-drugs (DMARDs). In more details, three patients were assuming leflunomide, 3 methotrexate in monotherapy and 2 methotrexate plus an anti-TNF-α biological drug.

Patients with SSc-sSS presented a limited variant of the disease with a positivity for ACA detected in five cases out of seven. Only one patient had a past history of interstitial lung disease. At the time of the enrollment, all the patients showed only mild SSc related clinical manifestations (that is, sclerodactily, Raynaud's phenomenon, telangiectasias, eosophageal involvement, arthralgias and digital ulcerations) requiring only low-dose steroids, calcium channel blockers, prostacyclin analogues, prokinetic agents and/or proton pump inhibitors. None of the SSc-sSS patients had active lung interstitial disease or pulmonary hypertension and none was using immunosuppressive drugs when the salivary samples were collected.

In the second part of the study, the independent set cohort included 75 unselected, consecutive subjects: 21 healthy volunteers (49.1 ± 9.8 years, mean age ± SD), 19 patients with a diagnosis of pSS (52.5 ± 14.3 years, mean age ± SD), 10 patients with non-SS sicca syndrome (52.7 ± 17.9 years, mean age ± SD) and 25 secondary-SS (8 RA-sSS (59 ± 10.9 years, mean age ± SD) and 17 SSc-sSS (51 ± 14.8 years, mean age ± SD). Demographic and glandular manifestations of the validation cohort did not differ from the derivation cohort of the subjects included in the first phase of the study.

### Saliva collection and processing

Between 1 and 3 ml of saliva were obtained from each subject. No differences were detected in the mean volume of saliva collected from each group of patients. In particular, we obtained a mean volume of 1.05 ± 0.77 ml of saliva from patients with pSS, 1.02 ± 0.78 ml from patients with non-SS sicca symptoms and 1.06 ± 0.49 ml and 1.07 ± 0.34 from patients with RA-sSS and SSc-sSS respectively.

### Identification of differentially expressed salivary proteins in pSS with respect to healthy, RA-sSS, SSc-sSS and non-SS sicca syndrome

**Figure 1:**
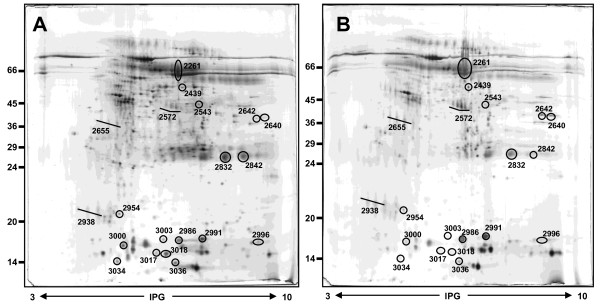
**Representative 2D map of primary Sjögren's syndrome (pSS) (A) and healthy subject (B)**. A total of 150 µg of whole saliva proteins was separated by 2D using 18 cm pH 3 to 10 L strip (IPG) and 12.5% SDS-PAGE. Proteins were detected by silver staining. The map was analysed by Image master 2D platinum software. Spot numbers indicate all the proteins identified by MS/MS and refer to the number reported in Tables 2-4.

**Figure 2:**
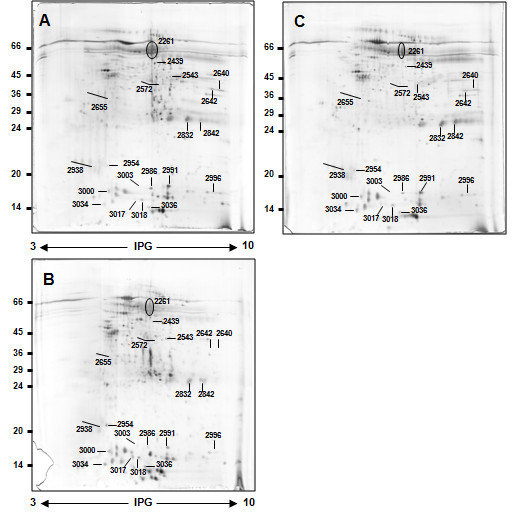
**Representative 2D map of secondary Sjögren's syndrome and other sicca syndromes**. (A) non-SS sicca syndrome, (B) secondary Sjögren's syndrome concomitant to rheumatoid arthritis RA-sSS and (C) secondary Sjögren's syndrome concomitant to systemic sclerosis SSc-sSS whole saliva proteins. A total of 150 µg of proteins was separated by 2D using 18 cm pH 3 to 10 L strip (IPG) and 12.5% SDS-PAGE. Proteins were detected by silver staining. The map was analysed by Image master 2D platinum software. Spot numbers indicate all the proteins identified by MS/MS and refer to the number reported in Tables 2-4.

Typical 2DE gel images of exemplary gels of salivary protein extracts from pooled samples of healthy volunteers, pSS patients, RA-sSS, SSc-sSS and non-SS sicca syndrome subjects are shown in Figures 1 and 2. By computational gel image comparison, a total of 28, 6, 7 and 12 protein spots were found to be differentially expressed in healthy volunteers, non-SS sicca syndrome, SSc-sSS and RA-sSS salivary profiles, with respect to pSS samples. For "representative" salivary proteins, the identification was obtained by direct comparison with images of previous studies. For unknown proteins, we proceeded with MS/MS analysis. Of the 15 cut spots, 80% were identified by MS/MS spectrometry, yielding 9 different protein identifications. A list of identified proteins, MW, isoelectric point (pI) score and coverage values of MS/MS is shown in Table 2.

**Table 2:** MS/MS data of differential protein spots from pSS, non-SS sicca syndrome and sSS syndromes

Spot No	Accession No ^a^	Protein name	Gene name	Theoretical Mr ^b^	Theoretical pI ^b^	Matched peptides ^c^	Coverage (%)	Peptides identified ^d^
**2261**	P04745	α-amylase 1	*AMY1A*	58	6.47	16	43	RSGNEDEFRN
**2842**	Q6GMW1	IGKC protein	*IGKC*	26	6.9	8	33	KSFNRGE
**2439** **2543**	P06733	α-enolase	*ENO1*	47	7.01	8	25	RNFRNPLAK
**2640** **2642**	P04406	G3PDH	*GAPDH*	36	8.57	3	9	KAGAHLQGGAKRV
**3017** **3034**	P06702	Protein S100-A9	*S100A9*	13	5.71	4	43	RLTWASHEKM
**2996**	P01034	Cystatin-C	*CST3*	16	9.00	6	49	KASNDMYHSRA
**3018**	P31151	Protein S100-A7	*S100A7*	11	6.27	3	26	RSIIGMIDMFHKY
**2832**	A0N5G5	Rheumatoid factor D5 light chain (Fragment)	*V<kappa>3*	13	9.15	2	23	RLLIYDASNRA
**2954**	P31025	Lipocalin-1	*LCN1*	19	5.39	3	27	RGLSTESILIPRQ

a) Accession number was derived from the ExPASy database. b) Theoretical molecular weight (kDa) and pI from the ExPASy database. c) The number of peaks which match to the trypsin peptides. d) Each spot corresponding to one certain protein had at least one of the shown peptides identified.

G3PDH, glyceraldehydes-3-phosphate dehydrogenase; IGKC: immunoglobulin k light chain; pSS, primary Sjögren's syndrome; SS: Sjögren's syndrome; SSc-sSS secondary Sjögren's syndrome concomitant to systemic sclerosis; sSS: secondary Sjögren's syndrome.

Table 3 summarises the most significant differences among pSS patients, healthy volunteers and non-SS sicca syndrome in the expression of both "representative" and newly identified salivary proteins. In addition, the fold changes of the expression of previously and newly identified proteins in RA-sSS and SSc-sSS subjects with respect to pSS, and the relative *P*-values, are shown in Table 4. The proteins found to be differentially expressed are also circled in the representative gels (Figures 1 and 2).

**Table 3:** Differential protein spots from pSS, healthy volunteers and non-SS sicca syndrome patients (*P *<0

Spot No	Accession No	Protein name	Gene name	Mean ± SD (pSS)	Mean ± SD (healthy)	Mean ± SD (sicca non-SS)	Fold of variation pSS *vs *healthy	Fold of variation pSS *vs *sicca non-SS
**2261**	P04745	α-amylases precursor	*AMY1A*	2.58 ± 0.44	4.77 ± 0.39	4.24 ± 0.67	-1.8**	-1.6**
**2572**	Q5FC00	Carbonic anhydrase VI	*CA6*	0.65 ± 0.11	1.50 ± 0.47	1.07 ± 0.26	-2.3*	-1.6*
**2642**	P04406	G3PDH	*GAPDH*	0.07 ± 0.03	0.31 ± 0.03	0.23 ± 0.85	-4.4***	-3.2*
**2543**	P06733	α-enolase	*ENO1*	0.08 ± 0.02	0.04 ± 0.01	0.05 ± 0.03	+2**	+1.6
**2842**	Q6GMW1	IGKC	*IGKC*	1.33 ± 0.49	0.17 ± 0.30	0.54 ± 0.42	+7.8*	+2.5
**3018**	P31151	Protein S100-A7 (Psoriasin)	*S100A7*	0.47 ± 0.21	0.09 ± 0.04	0.32 ± 0.15	+5.2*	+1.5
**3000**	P06702	Protein S100-A9 (Calgranulin B)	*S100A9*	2.98 ± 0.10	2.00 ± 0.42	2.75 ± 0.54	+1.5*	ns
**2986**	P01037	Cystatin SN precursor	*CST1*	0.91 ± 0.36	2.37 ± 0.01	0.84 ± 0.44	-2.6**	ns
**2938**	P12273	PIP	*PIP*	0.05 ± 0.08	0.33 ± 0.01	0.09 ± 0.17	-6.6**	-1.8
**2655**	Q96DR5	SPLUNC-2	*SPLUNC2*	0.04 ± 0.07	0.40 ± 0.17	0.40 ± 0.18	-10*	-10*
**3003**	Q01469	E-FABP	*FABP5*	0.17 ± 0.07	0.02 ± 0.03	0.06 ± 0.05	+8.5*	+2.8*
**3036**	P61769	β-2 microglobulin	*B2M*	0.39 ± 0.04	0.17 ± 0.04	0.23 ± 0.05	+2.3**	+1.7

NB, values that are significantly different from pSS (*P <0.05, **P <0.01, ***P <0.001) as determined by statistical analysis. E-FABP, epidermal fatty acid binding protein; G3PDH, glyceraldehydes-3-phosphate dehydrogenase; IGKC, immunoglobulin k light chain; PIP, prolactin-inducible protein precursor; pSS, primary Sjögren's syndrome; SPLUNC-2, lung and nasal epithelium clone 2; SS, Sjögren's syndrome

**Table 4:** Differential protein spots in pSS, RA-sSS and SSc-sSS (*P *<0

Spot No	Accession No	Protein name	Gene name	Mean ± SD (pSS)	Mean ± SD (RA- sSS)	Mean ± SD (SSc-sSS)	Fold of variation pSS *vs *RA- sSS	Fold of variation pSS *vs *SSc-sSS
**3017**	P06702	(Protein S100-A9) Calgranulin B	*S100A9*	0.15 ± 0.07	0.37 ± 0.07	-	-2.46*	-
**3034**	P06702	(Protein S100-A9) Calgranulin B	*S100A9*	0.20 ± 0.09	0.56 ± 0.17	-	-2.8*	-
**2439**	P06733	α-enolase	*ENO1*	0.13 ± 0.02	0.05 ± 0.02	-	+2.6**	-
**2543**	P06733	α-enolase	*ENO1*	0.08 ± 0.02	0.19 ± 0.03	-	-2.4**	-
**2842**	Q6GMW1	IGKC	*IGKC*	1.33 ± 0.49	0.05 ± 0.09	-	+26*	-
**2832**	A0N5G5	Rheumatoid factor D5 light chain	*V<KAPPA>3*	1.97 ± 0.11	0.29 ± 0.26	-	+6.8***	-
**2954**	P31025	Lipocalin	*LCN1*	0.06 ± 0.05	0.37 ± 0.07	-	-6.2**	-
**3036**	P61769	β-2 microglobulin	*B2M*	0.39 ± 0.04	0.25 ± 0.04	0.25 ± 0.03	+1.6*	+1.6*
**2642**	P04406	G3PDH	*G3PDH*	0.07 ± 0.03	-	0.19 ± 0.10	-	-2.7*
**2996**	P01034	Cystatin-C	*CST3*	0.21 ± 0.08	-	0.05 ± 0.04	-	-4.2*

NB, values that are significantly different from pSS (*P <0.05, **P <0.01, ***P <0.001) as determined by statistical analysis.

G3PDH, glyceraldehydes-3-phosphate dehydrogenase; IGKC, immunoglobulin k light chain; pSS, primary Sjögren's syndrome; RA-sSS, secondary Sjögren's syndrome concomitant to rheumatoid arthritis; SSc-sSS, secondary Sjögren's syndrome concomitant to systemic sclerosis

As described in Table 3, 12 proteins were differently expressed in pSS with respect to healthy volunteers. Among them, six proteins showed a >1.5-fold of increase in pSS (that is, calgranulin B [UniProtKB: P06702], β-2 microglobulin [UniProtKB:P61769], epidermal fatty acid binding protein (E-FABP) [UniProtKB:Q01469], psoriasin [UniProtKB:P31151], IGKC protein ([UniProtKB:Q6GMW1] and α-enolase (47 KDa) [UniProtKB:P06733]) and six were characterised by a >1.5 fold decrease (that is, α-amylases precursor [UniProtKB:P04745], carbonic anhydrase VI [UniProtKB:Q5FC00], cystatin SN precursor [UniProtKB:P01037], prolactin-inducible protein precursor (PIP) [UniProtKB:P12273], short palate, lung and nasal epithelium clone 2 (SPLUNC-2) [UniProtKB:Q96DR5], glyceraldehydes-3-phosphate dehydrogenase (G3PDH) [UniProtKB:P04406]). When pSS patients were compared to patients affected by non-SS sicca syndrome, α-amylases precursor, carbonic anhydrase VI, G3PDH and SPLUNC-2 were still significantly decreased in the former group, while, on the contrary E-FABP, β-2 microglobulin and IGKC protein showed a persistent, significant increase. Three more proteins were differently expressed in pSS with respect to sSS (Table 4): lipocalin [UniProtKB:P31025] and rheumatoid factor D5 light chain [UniProtKB:A0N5G5] which were respectively increased and decreased in RA-sSS, and Cystatin C [UniProtKB:P01034], which was significantly reduced in SSc-sSS. No further differences were documented between pSS and sSS with the exception of β-2 microglobulin, which was increased in pSS in comparison to both RA-sSS and SSc-sSS, IGKC protein which was significantly decreased in RA-sSS and G3PDH, which was decreased in SSc-sSS. Finally, we observed that single spots of the same proteins were characterised by a different optical density in pSS-patients and controls. From this point of view, we found that the spots n° 2543 (47 KDa α-enolase), n° 3034 and 3017 (calgranulin B) were significantly increased in RA-sSS while the spot n° 2439 (59KDa α-enolase) was significantly decreased in RA-sSS in comparison to pSS.

### Principal component analysis (PCA) of match set data

**Figure 3:**
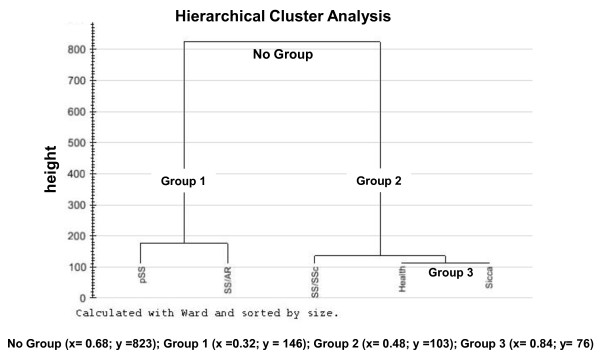
**Dendogram obtained after PCA**. The dendrogram is a graphical representation of the results of hierarchical cluster analysis. This is a tree-like plot where each step of hierarchical clustering is represented as a fusion of two branches of the tree into a single one. The branches represent clusters obtained on each step of hierarchical clustering. A close relationship was observed between primary Sjögren's syndrome (pSS) and patients with secondary Sjögren's syndrome (sSS) concomitant to rheumatoid arthritis (RA-sSS) classes while sicca syndrome and healthy volunteers appeared to be more similar. The protein profile of patients with secondary Sjögren's syndrome (sSS) concomitant to systemic sclerosis (SSc-sSS) was placed at an intermediate level between the previously mentioned clusters.

Figure 3 shows the dendogram obtained after the PCA application to the entire data of the match sets, including healthy, pSS, non-SS sicca syndrome and RA-sSS and SSc-sSS subjects. The vertical axis indicates the loss in within-cluster similarity, that is, the variance increase when the clusters are merged. A close relationship was observed between pSS and RA-sSS (Group 1), while on the other hand non-SS sicca syndrome and healthy volunteers appeared to be more similar to each other (Group 3). The protein profile of SSc-sSS patients was placed at an intermediate level between the previously mentioned clusters, and created a different group together with non-SS sicca syndrome and healthy volunteers (Group 2).

### Validation of β-2 microglobulin, IGKC and α-enolase protein by WB analysis and ELISA

**Figure 4:**
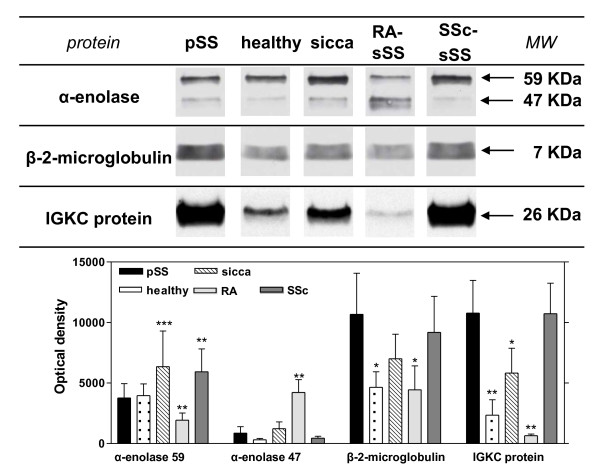
**Validation of α-enolase, β-2 microgloblulin and immunoglobulin k light chain (IGKC)**. **(A) **Conventional SDS gels were run with protein extracts from mix pooled WS samples of primary Sjögren's syndrome (pSS), non-SS sicca syndrome, RA-sSS and SSc-sSS syndromes patients using 12% resolving capacity. Twenty-five micrograms of total proteins were loaded into each lane for each sample. Proteins were transferred onto nitrocellulose membranes and incubated with specific antibodies (dilution 1:200 for anti-α-enolase and anti-β-2 microgloblulin; dilution 1:10,000 for anti-IGKC) against the target proteins. **(B) **Densitometry of the blots was performed and the bar graph shows the mean ± SD of the optical density values of three independent experiments. Values that are significantly different from pSS (*P <0.05, **P <0.01, ***P <0.001) value as determined by statistical analysis.

A representative immunoblot of β-2 microgloblulin, α-enolase and IGKC and the resulting mean optical density values ± SD is represented in Figure 4 (Figure 4, bar graphs). As shown in the figure, WB analysis confirmed a significant increase of β-2 microglobulin (*P-*value = 0.02) and IGKC protein (*P *= 0.01) in pSS with respect to healthy volunteers while documented only a trend in favour of the increased of α-enolase (47 KDa) (pSS vs healthy volunteers, *P *= 0.08). In fact, β-2 microglobulin (*P *<0.001) and α-enolase (47KDa) (*P *= 0.002) were both found statistically significant in pSS vs healthy volunteers at the ELISA test (Table 5). WB also confirmed a significant increase of IGKC protein in pSS with respect to RA-sSS (*P *= 0.01, WB).

**Table 5:** Mean values of salivary marker proteins

	β-2 microglobulin mean ± SD (μg/ml)	α-enolase mean ± SD (ng/ml)	α-amylase mean ± SD (U/mg of proteins)
**pSS**	1.7 ± 0.5	82.4 ± 34.1	35.7 ± 17.5
**healthy**	0.8 ± 0.3	37.0 ± 27.3	34.5 ± 15.5
*P-value vs pSS *	*<0.001*	*0.002*	*0.893*
**sicca non-SS**	1.3 ± 0.7	74.2 ± 33.4	40.0 ± 18.7
*P-value vs pSS*	*0.08*	*0.191*	*0.538*
**RA- sSS**	1.5 ± 0.8	103.0 ± 15.4	35.3 ± 19.9
*P-value vs pSS*	*0.514*	*0.204*	*0.96*
**SSc-sSS**	1.5 ± 0.6	78.2 ± 31.6	38.0 ± 22.3
*P-value vs pSS*	*0.05*	*0.392*	*0.718*

pSS, primary Sjögren's syndrome; RA-sSS, secondary Sjögren's syndrome concomitant to rheumatoid arthritis; SSc, systemic sclerosis; SSc-sSS, secondary Sjögren's syndrome (sSS) concomitant to systemic sclerosis; sSS, secondary Sjögren's syndrome.

As far as the expression of β-2 microglobulin among pathological controls was related, discordant data were observed both at the WB and the ELISA tests. Overall, taking into account the 2DE data and the of WB and ELISA results, we hypothesised a trend in favour of increased β-2 microglobulin levels in pSS in comparison to both non-SS sicca syndrome (*P *= 0.11, WB; *P *= 0.087, ELISA test), RA-sSS (*P *= 0.02, WB; *P *= 0.51, ELISA test) and SSc-sSS (*P *= 0.53, WB; *P *= 0.05, ELISA test). Intriguingly, we also found a significant association between β-2 microglobulin (ELISA test) and anti-Ro/SSA antibodies (*P *= 0.008). As far as α-enolase protein was concerned, the resulting immunoreactive pattern confirmed the presence of two specific bands with a different level of intensity. The major immunoreaction was with a protein of 59 kDa, while the other was at 47 kDa. These results were in agreement with those obtained by 2DE/MS/MS analysis which documented two different spots (n° 2439 and n° 2543), both identified as α-enolase. In particular, the 47 kDa band (spot n° 2543) was expressed at the highest levels in RA-sSS (pSS *vs *RA-sSS *P-*value = 0.0013, WB) while the 59 kDa band (spot n° 2439) was significantly reduced (pSS *vs *RA-sSS *P-*value = 0.03, WB). At the ELISA test, α-enolase resulted as not differently expressed in RA-sSS and in pSS (*P *= 0.204); however, we found that α-enolase was still significantly increased in RA-sSS with respect to healthy volunteers (*P *= 0.001) and that α-enolase was significantly associated to rheumatoid factor (*P *= 0.03). No significant associations were found between ELISA test results and minor salivary gland biopsy focus score.

### The immune reactive pattern of α-amylases fragmentation

**Figure 5:**
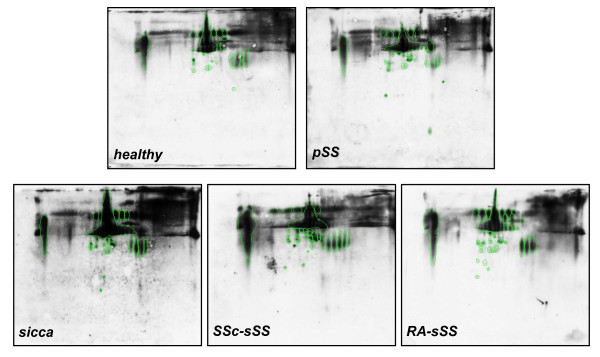
**Immunoblot of the α-amylase in pSS, non-SS sicca syndrome, RA-sSS and SSc-sSS syndromes**. The pattern of expression of α-amylases protein was investigated in different classes of patients by 2DE and subsequently by WB with specific antibody direct *versus *full length of human recombinant protein. Aliquots of 100 µg of proteins extracts from mix pooled WS samples of each class were separated by 2DE using 3 to 10 linear strips 13 cm before Western blot analysis. The dilution was 1:500 and 1:10,000 for anti-α-amylase primary antibody and anti-duck, respectively.

Regarding α-amylases fragmentation, in addition to the main spot of the α-amylases precursor, the immunoreactive pattern documented in pSS and sSS, a number of additional spots identified as α-amylases which were distributed in a large pI range with a different level of intensity suggesting a typical profile of protein fragmentation (Figure 5). These observations and the fact that enzymatic activity assay did not document any difference in the levels of the α-amylases among pSS patients and healthy or pathological controls (Table 5) allowed us to make the hypothesis that α-amylases fragmentation might be considered as the principal mechanism responsible for the decrease of the main spot of α-amylases precursor in pSS and sSS with respect to non-SS sicca syndrome and healthy volunteers.

### Network construction for biological processes

**Figure 6:**
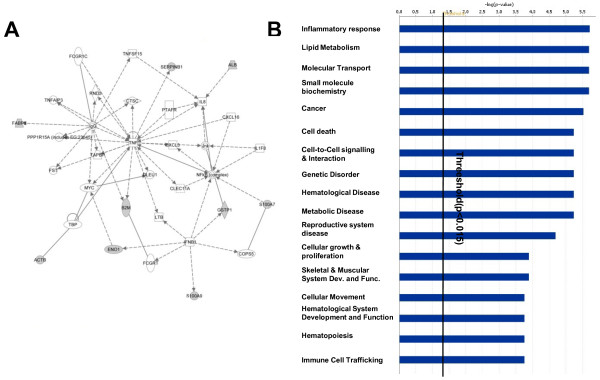
**Network analysis of differentially expressed proteins using the IPA software**. Proteins of WS that were found to be significantly different among pSS, non-SS sicca syndrome, RA-sSS and SSc-sSS syndromes were analysed by using IPA. **(A) **This network shows 35 proteins that work together for Cellular Movement, Haematological System Development and Function, Haematopoiesis. Ten of these 35 proteins were found in our salivary proteome. Protein-to-protein direct (solid lines) or indirect (dashed lines) interactions/regulations, based on information published in the literature are shown in the figure. In the panel **B**, the top 17 functions of the proteins identified in the network are shown. Proteins were matched to 72 functions with significance above 1.83 (*P *<0.015). The top 17 functions involving the 35 proteins are shown here with the number of associated proteins that were identified for each function. ACTB, actin beta; ALB, albumin, B2M, beta2microglobulin; CLEC11A, C-type lectin domain family 11 member A; COPS5, COP9 signalosome complex subunit 5; CTSC, cathepsin C; CXCL16, C-X-C motif chemokine 16; CXCL3, C-X-C motif chemokine 3; DLEU1, Leukemia-associated protein 1; ENO1, Alpha-enolase; FABP5, fatty acid-binding protein, epidermal; FCGR1C, putative high affinity immunoglobulin gamma Fc receptor IC; FCGRT, IgG receptor FcRn large subunit p51; FST, follistatin; GSTP1, glutathione S-transferase P; IFN-β1, interferon beta; IgG, immunoglobulin G; IL1F8, interleukin 1 family, member 8; IL8, interleukin 8; JNK, c-Jun N-terminal kinase; LNB, Lymphotoxin beta; MYC, v-myc myelocytomatosis viral oncogene homolog (avian); NFkB (complex), nuclear factor NF-kappa-B; PPP1R15A, protein phosphatase 1 regulatory subunit 15A; PTAFR, platelet-activating factor receptor; RND3, rho-related GTP-binding protein RhoE; S100A7, psoriasin 1; S100 A8, protein S100-A8; S100A9, protein S100-A9; SERPINB1, leukocyte elastase inhibitor; TAPBP, tapasin-related protein; TBP, TATA box binding protein; TNF, tumor necrosis factor; TNFAIP3, tumor necrosis factor, alpha-induced protein 3; TNFSF15, tumor necrosis factor ligand superfamily member

Three networks were generated by the Ingenuity software. The one with the highest score (score value 20) is shown in Figure 6. This network shows 35 proteins that work together for Cellular Movement, Haematological System Development and Function, Haematopoiesis, and 9 of these 35 proteins were found in our salivary proteome. Genes or gene products are represented as nodes, and the biological relationship between two nodes is represented as an edge (line). Figure 6 shows the direct or indirect relationships exhibited by the proteins with each other within the network by using solid and dashed lines respectively. The figure clearly shows that TNF, nuclear factor-kappa B (NF-kB) and IgG are key nodes within the network. These key gene nodes are connected directly and indirectly to many of the putative salivary protein biomarkers identified in pSS. The identified proteins included: E-FABP, β-2 microglobulin, calgranulin B (protein S100-A9), psoriasin (protein S100-A7), and α-enolase. The IPA demonstrated that the major involved biological functions resulted in the following categories: inflammatory response pathways, lipid metabolism processes, molecular transport, immune cell trafficking, cancer and haematological disease categories.

## Discussion

The overall results of this study allowed us to verify and pre-clinically validate in a large cohort of patients a number of potential discriminatory biomarkers in the whole saliva of pSS, in comparison to both healthy volunteers and pathological controls. In this study we found that the pSS salivary profile was characterised by a decrease in many secretory proteins (that is, α-amylases precursor, carbonic anhydrase VI, PIP, SPLUNC-2, G3PDH, and cystatins), an increase of proteins related to the autoimmune response (that is, β-2 microglobulin, IGKC protein, and rheumatoid factor D5 light chain) and an increase of proteins related to systemic and local inflammation (α-enolase, lipocalin and S100-A7 and A9 proteins). On the other hand, as expected, subjects with RA-sSS or SSc-sSS shared a number of salivary biomarkers, whereas subjects with sicca complaints but without SS were characterised by a proteomic profile much closer to that of healthy subjects with the exception of an increase of proteins related to inflammation (that is, S100 proteins) and a decrease of secretory proteins, such as PIP and SPLUNC-2. In fact, the latter was still significantly increased in patients with non-SS sicca with respect to pSS patients while PIP showed a one-fold variation when compared to pSS. The similarities and the differences between the groups were clearly displayed in the dendogram representation. The dendogram showed, in particular, that pSS and RA-sSS were so close in value that together they made a cluster easily distinguishable from the other groups. Subjects with SSc-sSS created a second, different cluster which was in between the others, showing not only a reduction of many acinary proteins and an increase of proteins related to lymphocyte activation, but also an increased expression of G3PDH which was closer to the profiles of non-SS sicca syndrome patients and healthy volunteers. In turn, healthy volunteers and patients with non-SS sicca syndrome were apparently very similar and represented a third cluster.

These results confirmed our previous preliminary data [12] and pre-existing literature [13-17]. However, in this study, the expression of salivary biomarkers was also verified in an independent cohort of healthy volunteers and pathological controls, distinct from the derivation cohort, by using additional antibody detection tests (WB and ELISA). This allowed us to conclude that a panel of candidate biomarkers rather than a single specific protein may apparently be able to better distinguish pSS from healthy volunteers and other pathological disorders.

In the present study, we first described 15 proteins which could represent candidate biomarkers to be included in a potential diagnostic panel for pSS. Some of them appeared to be significantly increased (that is, psoriasin) or decreased (that is, cystatin SN, PIP) only in comparison with healthy volunteers and, therefore, their diagnostic role for pSS remains controversial. On the contrary, out of the identified 15 proteins, α-amylases precursor, β-2 microglobulin, G3PDH, IGKC protein, E-FABP, carbonic anhydrase VI and SPLUNC-2 showed the most significant differences in the expression in comparison both to healthy volunteers and non-SS sicca syndrome and, therefore, appeared to be the most significant discriminatory biomarkers for SS.

A role for these proteins of potential biomarkers for pSS and sSS has been already hypothesised by previous proteomic studies. In particular, Ryu *et al*. [13] demonstrated a significant increase of β-2 microglobulin and IGKC protein and a reduction of α-amylases precursor and carbonic anhydrase VI in the stimulated parotid saliva obtained from 41 primary SS patients. A significant reduction of α-amylases in pSS was also reported by Peluso and co-workers [14]. In the present work, we confirmed the significant decrease of the main spot of α-amylases precursor in pSS and sSS with respect to healthy volunteers and non-SS sicca syndrome. We also found that the pSS salivary profile was characterised by a peculiar abundance of α-amylases precursor fragments. We concluded that to a certain extent, the decrease of this acinar protein might be explained by pSS fragmentation processes which have been previously reported as related to an unbalanced expression of proteases and proteases inhibitors [12]. We also documented an increase of IGKC protein, rheumatoid factor D5 light chain and β-2 microglobulin. As far as β-2 microglobulin is related, Hu S and coworkers [15,17] identified and preclinically validated β-2 microglobulin, cathepsin D, and α-enolase as potential biomarkers for pSS. In the current study we were unable to identify cathepsin D. However, in line with the studies by Hu S *et al*. [15,17] we found that β-2 microglobulin was significantly increased in pSS. WB and ELISA tests confirmed a trend in the expression of the protein with the highest levels in pSS and the lowest levels in healthy volunteers and a significant association between β-2 microglobulin and antiRo/SSA positivity was also found. The increased expression of this protein in pSS whole saliva might thus reflect both the systemic B-cells activation and the increased intra-glandular immunoglobulin synthesis, which are peculiar aspect of the disease [23-25].

Finally, we also documented an increase in the salivary expression of α-enolase in pSS in comparison to healthy controls. In addition, we also found that α-enolase (47 KDa) was significantly higher in patients with RA-sSS with respect to pSS and significantly associated to the positivity for rheumatoid factor (*P *= 0.03). Similar trends were also reported for lipocalin and for many spots of calgranulin B (S100-A9) which showed the highest levels in RA-sSS patients. Intriguingly, since increased levels of all these proteins have been already reported in biological fluids of patients with RA without sSS [26,27], we speculated that proteomic analysis of whole saliva could represent a novel technique not only for the diagnosis of disorders involving salivary glands but also for systemic autoimmune disorders even in the absence of any salivary exocrinopathy. In line with this work hypothesis, we also found, in this study, that the salivary profile of patients with SSc-sSS, was characterised by a significant increase of G3PDH, which has been already described in whole saliva of patients with SSc without sSS [28].

Moreover, in addition to the mentioned potential diagnostic applications, our results support the hypothesis that the entire proteome of saliva might mirror the disease process and provide useful insights into the pathogenesis of the lesions helping to monitor the evolution of the disease process over time. The potential impact of the proteomic approach in the pathogenetic research of autoimmune diseases might be emphasized if we employ a novel statistical platform, the IPA Knowledge *base*, to the analysis of biomarkers signatures in pSS. We found that many of the involved candidate biomarkers could be associated in a network which was able to mirror many of the several pathways involved in the pathogenesis of pSS. In this study, the biological functions that received the highest score of association with the protein panels were represented by cellular trafficking and movement, cell-to-cell interaction, inflammatory response, cell death, haematological system function and haematological cancers. Interestingly, all these biological processes are known to take part in many of the pathophysiological steps of the disease from the gland infectious and/or immune-mediated attack to lymphoproliferation [29,30]. Looking at the nodes of the network, which were represented by interferon-β (IFN-β), the interleukin-8 (IL-8), the tumour necrosis factor-α (TNF-α) and the NF-kB many potential speculations might arise. For example, an enhanced activity of the type-I IFN system (and to a lesser extent of type-II IFN system) has been associated to the early stages of pSS pathogenesis, and can be considered the link between the disease onset and an hypothetical environmental trigger (that is, infectious agent) in genetically predisposed subjects [29,30]. Moreover, an increased expression of IFN-regulated genes has been described in pSS salivary glands [15] and correlated with the production of anti-Ro/SSA and anti-La/SSB auto-antibodies and with the up-regulation of numerous INF-induced chemokines (that is, CXCL10, CXCL13) [31-35]. Finally, IL-8 is involved mainly in cellular trafficking and defence, while TNF-α and NF-kB are important inducers of inflammatory response and apoptosis, and may amplify the autoimmune deregulation contributing to the apoptotic death of epithelial cells, to ductal hyperplasia and glandular fibrosis, all recognised hallmarks of pSS [30,34].

## Conclusions

In conclusion, this study demonstrated that novel, non-invasively-collected salivary proteomic biomarkers might be helpful for an early and accurate characterisation of pSS and sSS. We focused the attention on a panel of seven proteins which were at the same time differently expressed in pSS, non-SS sicca syndrome and healthy volunteers. In addition, we found that some of the sSS identified biomarkers apparently reflected not only the SS component but also the concomitant systemic autoimmune disorders shedding new light on the potential diagnostic role of saliva in autoimmune diseases irrespectively from salivary glands involvement. Of course, this study has some limitations: 2DE gels were obtained by pooling the salivary samples of each group, WB and ELISA tests were performed only for α-enolase, β-2 microglobulin, and α-amylases. However, this study soundly confirmed that saliva might have a great potential as a novel biofluid for the diagnosis of systemic autoimmune disorders with definite advantages in terms of non-invasiveness and reproducibility. Finally, in this study salivary proteomics also appeared to be useful in exploring the pathogenetic pathways of different systemic disorders. It would be advisable, as a potential development of this work, to set up, in the near future, different strategies to measure easily the single components of the identified proteomic salivary panel in single patients in order to develop a multivariate prediction model able to improve a screening diagnostic process for pSS in clinical practice. This will allow the clinician to take full advantage of the many potentialities of salivary fluid as an ideal milieu for the diagnosis of pSS and other systemic diseases and for the identification of the pathogenetic processes underlying glandular and systemic disorders.

## Abbreviations

2DE, two-dimensional electrophoresis; ACA, anticentromere auto-antibodies; AECG, International Classification Criteria for the disease; anti-CCP, anti-cyclic citrullinated peptide; CCP, cyclic citrullinated peptide; CHAPS, 3-[(3-cholamidopropyl)dimethylammonio]-1-propanesulfonate; DAS28, Disease Activity Score 28; DMARDs, disease-modifying antirheumatic-drugs; DTT, dithiothreitol; E-FABP, epidermal fatty acid binding protein; ELISA, enzyme-linked immunosorbent assay; ENA, extractable nuclear antigen; G3PDH, glyceraldehydes-3-phosphate dehydrogenase; IFN-β, interferon-β; IGKC, immunoglobulin k light chain; IL-8, interleukin-8; IPA, Ingenuity Pathway Analysis; MALDI-TOF-MS, matrix-assisted laser desorption/ionisation time-of-flight mass spectrometry; NF-kB, nuclear factor-kappa B; PCA, principal component analysis; PIP, prolactin-inducible protein precursor; pSS, primary Sjögren's syndrome; RA, rheumatoid arthritis; RA-sSS, patients with sSS concomitant to RA; SPLUNC-2, lung and nasal epithelium clone 2; SS, Sjögren's syndrome; SSc, systemic sclerosis; SSc-sSS, patients with sSS concomitant to SSc; sSS, secondary Sjögren's syndrome; TNF-α, tumour necrosis factor-α; WB, Western blot; WS, whole saliva

## Competing interests

The authors declare that they have no competing interests.

## Authors' contributions

AL and SB conceived of the study and drafted the manuscript. CB collected samples and participated in prognosis analysis, conceived of the study and drafted the manuscript. FC, YDV and ED carried out the proteomic studies and Western blot analysis. LG carried out the proteomic studies and Western blot analysis, performed image analysis and drafted the manuscript. CG, FS and LB collected samples and participated in prognosis analysis. GG performed image analysis. All the authors read and approved the final manuscript for publication.
